# Primary localized retroperitoneal sarcomas: report from Slovenian sarcoma referral center

**DOI:** 10.1186/s12957-020-02038-9

**Published:** 2020-10-27

**Authors:** Marko Novak, Andraž Perhavec, Milena Kerin Povšič, Matej Arnuš, Darja Eržen

**Affiliations:** 1 Department of Surgical Oncology, Institute of Oncology Ljubljana, Zaloška 2, 1105 Ljubljana, Slovenia; 2 Department of Anesthesiology and Intensive Care, Institute of Oncology Ljubljana, Zaloška 2, 1105 Ljubljana, Slovenia

**Keywords:** Retroperitoneal sarcoma, Referral center, Surgery, Survival

## Abstract

**Background:**

Sarcoma patients should be treated in high volume referral sarcoma centers. Compartmental resection is proposed as the best treatment option in retroperitoneal sarcoma patients.

**Methods:**

Institute of Oncology Ljubljana is the only referral sarcoma center in Slovenia. Having a population of 2.1 million poses a unique situation. We manage all sarcoma patients in the country and operate on patients with soft tissue tumors of extremities, trunk, and abdomen. Data for all consecutive patients surgically treated from January 1999 to December 2018 for primary localized retroperitoneal sarcoma was extracted from a prospective surgical database. Data about the incidence of sarcoma patients in Slovenia was extracted from the Cancer Registry of Republic of Slovenia. Clinicopathologic variables and the outcome were analyzed.

**Results:**

In total, 89 patients were included in the study. Median age was 62 years. Dedifferentiated liposarcoma was the most common histology (38.2%). Median tumor size was 21 cm. Compartmental resection was performed in 47.2% (42/89). Postoperative complication grade 3a or higher according to Clavien-Dindo classification had 30.3% (27/89) of patients. The 30-day and 90-day mortality rate was 2.2% and 5.6%. Median follow-up was 62.1 months. Corresponding 5-year overall survival was 67.2%, 5-year disease-specific survival was 72.6%, and 5-year local recurrence-free survival was 81.5%, respectively.

**Conclusion:**

Results from our institution show that referral sarcoma centers may achieve very good results in management of retroperitoneal sarcoma patients, despite not meeting the criteria for high volume hospitals, as long as they have multidisciplinary team, appropriate facilities, and expertise.

## Background

Surgery is the mainstay of treatment in primary localized retroperitoneal sarcomas (RPS). Compartmental resection offers the best chance for local control and/or potential cure to the patients [1, 2]. This approach comprises an en bloc resection of tumor with kidney, colon, and psoas fascia or muscle. If other adjacent organs are infiltrated, they are resected en bloc as well comprising multivisceral resection. A benefit of preoperative radiotherapy is still under research; thus, it is not routinely recommended. The EORTC 62092 trial (STRASS) failed to demonstrate the benefit of preoperative radiotherapy in abdominal recurrence of RPS [3]. Final results and publications about the STRASS study are awaited. The role of chemotherapy in RPS has not been investigated in a randomized controlled trial. En bloc resections for retroperitoneal tumors have been performed at the Institute of Oncology Ljubljana since 1975 [4, 5]. Primary aim of the study was to analyze the quality of surgery and the outcome of RPS patients treated at our institution in the last two decades. Secondary aim was to analyze the same parameters comparatively for each decade.

## Methods

Institutional Review Board (KSOPKR-0020/2020) and Ethical Committee (ERIDEK-0023/2020) approved the study. Clinicopathologic and follow-up data for all consecutive patients surgically treated for primary localized RPS at our institution from January 1999 to December 2018 was extracted from a prospective surgical database. Data about the incidence of sarcoma patients in Slovenia was extracted from the Cancer Registry of Republic of Slovenia.

Primary end point of the study was to investigate the quality of surgery in the last two decades by analyzing surgical resection margins, duration of surgery, blood loss, resection type, complication rates, and 30-day and 90-day postoperative mortality, and to analyze overall survival (OS), disease-specific survival (DSS), and local recurrence-free survival (LRFS). In the analysis of LRFS, deaths without evidence of disease and distant metastases (DM), whichever occurred first, were regarded as competing events. Concomitant local recurrence (LR) and DM were not included in the estimation of LRFS.

**Fig. 1:**
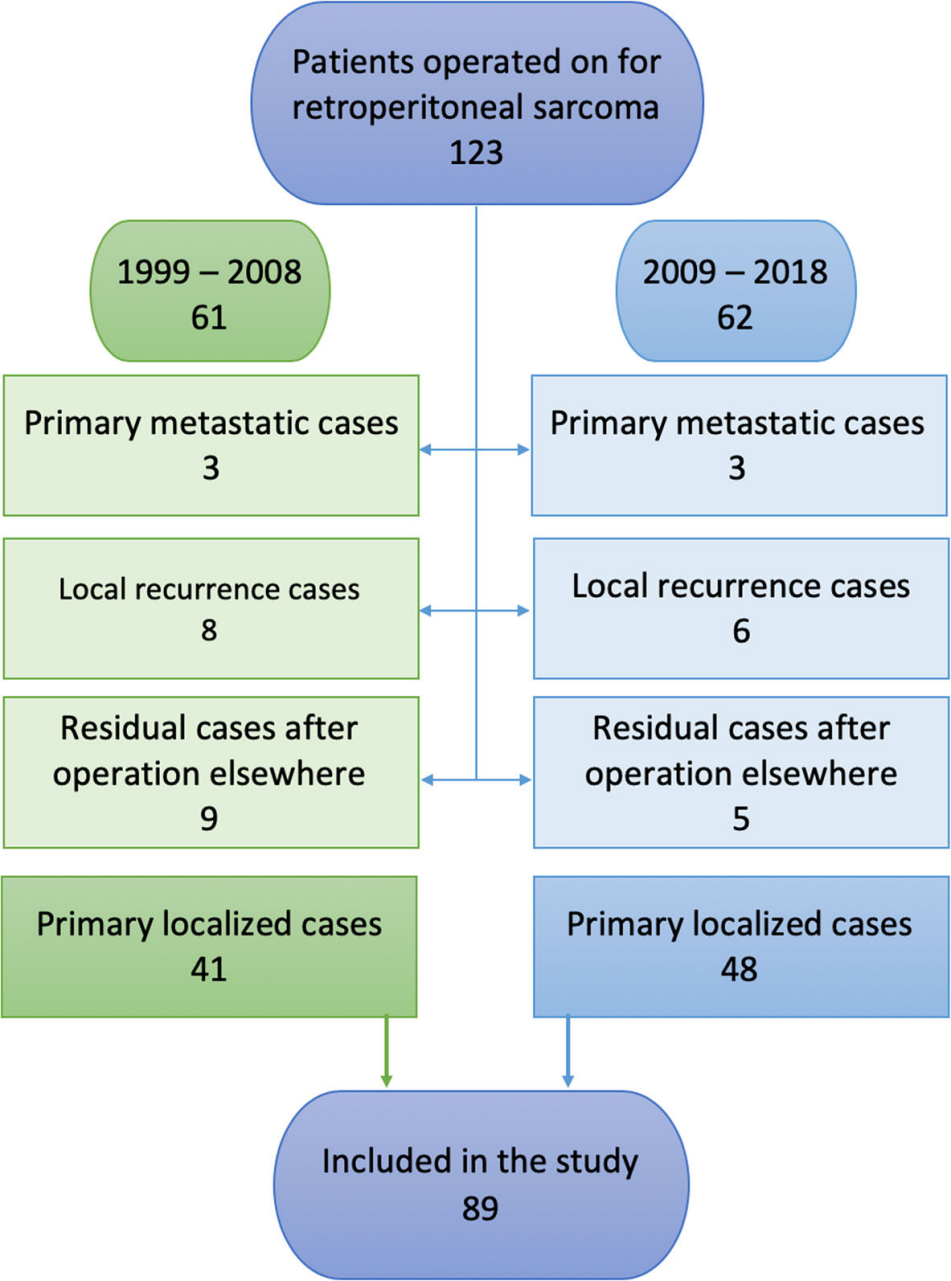
Flowchart. Patients with primary localized retroperitoneal sarcoma included in the study

Surgical devices of the modern era enable more meticulous hemostasis and shorter operation time. There was also a change of generations of sarcoma surgeons at our institution during this technological development. For those reasons, we decided to investigate the same parameters comparatively for each decade as a secondary end point. The cohort was divided in two groups. Flowchart in Fig. 1 presents the process of patient selection. In the first group were those who underwent surgery in the period from 1999 to 2008, and in the second group those who underwent surgery in the period from 2009 to 2018. All cases were presented at the multidisciplinary sarcoma team (MDT) before treatment.

Clinical characteristics were summarized using frequencies and percentages for categorical variables and median and range for continuous variables. Chi-square test was used to compare categorical variables and *t* test for continuous variables. Survival curves were estimated by Kaplan-Meier method and compared by the log-rank test. Results were considered statistically significant if two-sided *p* value < 0.05 was achieved. Statistical analysis was carried out using SPSS version 25.

## Results

There were 123 patients operated on for RPS at our institution in the study period. Patients with residual, recurrent, or primary metastatic disease at the referral among them were excluded from the analysis. In total, 89 patients with primary localized RPS were included in the study (Fig. 1). Median age of the patients was 62 years. At the referral, tumor was palpable in half of the cases (49.4%, 44/89) and 43.8% (39/89) of patients lost weight. In 25.8% (23/89), the tumor was coincidental finding. In the entire series, only 3 patients (3.4%) were operated on without the biopsy, 62.9% (56/89) had a fine needle aspiration, 23.6% (21/89) had core needle biopsy, and 10.1% of patients (9/89) had fine needle aspiration and core needle biopsy. Dedifferentiated liposarcoma was the most common histology (38.2%, 34/89). Median tumor size was 21 cm. Macroscopic complete resection (R0/R1) was achieved in all patients with microscopic negative margins in 76.4% (68/89). Compartmental resection was performed in 47.2% (42/89) and was extended into multivisceral resection in half of those cases (23.6%, 21/89). Only 2.2% (2/89) had the tumor removed without en bloc resection of any major organ. Organs were resected as follows: kidney in 57.3% (51/89), colon in 53.9% (48/89), adrenal in 42.7% (38/89), psoas fascia in 30.3% (27/89), psoas muscle in 28.1% (25/89), diaphragm in 20.2% (18/89), spleen and distal pancreas in 10.1% (9/89) each, inferior vena cava in 8.9% (8/89), and liver in 5.6% (5/89) of patients. Median number of resected organs per patient for the whole series was 4. Median hospital stay after surgery was 22 days. Postoperative complication grade 3a or higher according to Clavien-Dindo classification had 30.3% (27/89) of patients. Fifteen (16.9%, 15/89) required reoperation. The 90-day mortality rate was 5.6%. Reasons for reoperation and characteristics of patients who died within 90 days after surgery are summarized in Table 1.

**Table 1:** Morbidity for entire series and cause of death in five patients in 90-day postoperative period

Patient (*n* = 89)	%	Complication		
4	4.5	Postoperative bleeding		
4	4.5	Retroperitoneal abscess		
3	3.8	Abdominal abscess		
2	2.2	Anastomotic leak		
1	1.1	Intestinal gangrene		
1	1.1	Occlusion of iliac vessels		
Case (ASA)	Year of death	Complication	Cause of death	Time (days)
1 (3)	2011	Anastomotic leak	Sepsis, fulminant disease	55
2 (4)	2011	Tumor rupture, shock	Sepsis, DIC	10
3 (3)	2013	Retroperitoneal abscess	Sepsis, hepatorenal failure	79
4 (3)	2016	Abdominal abscess	Sepsis, cardiac decompensation	65
5 (3)	2018	Coronary stent occlusion	Intraoperative cardiac arrest	0

*ASA* American Society of Anesthesiologists classification, *DIC* disseminated intravascular coagulation

**Fig. 2:**
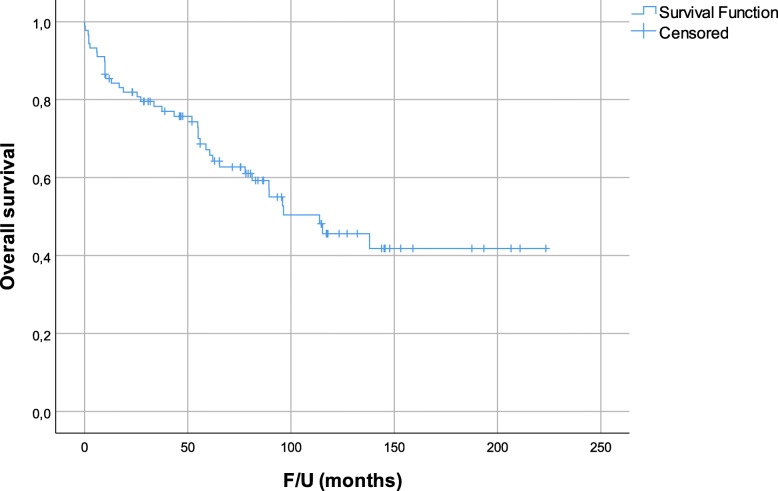
Overall survival for all patients

**Fig. 3:**
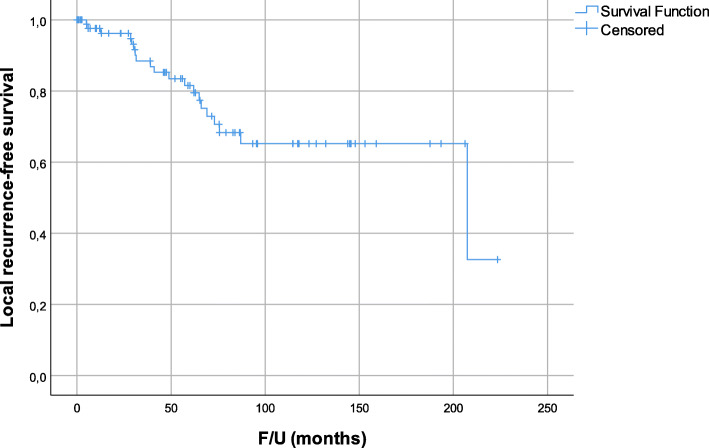
Local recurrence-free survival for all patients

Median follow-up from surgery was 62.1 months. In total, 39 patients died. The corresponding 5-year OS and DSS were 67.2% and 72.6% (Fig. 2). Twenty-five (28.1%) patients developed LR. Fourteen (15.7%) patients had LR only, 3 patients had DM followed by LR, 6 patients had LR followed by DM, and 2 patients had concomitant LR and DM. The corresponding 5-year LRFS was 81.5% (Fig. 3).

**Fig. 4:**
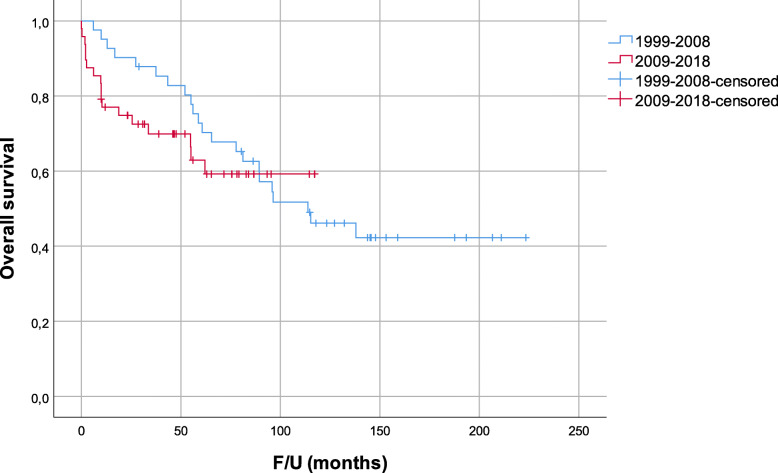
Overall survival divided by the period (*p* = 0.510)

**Fig. 5:**
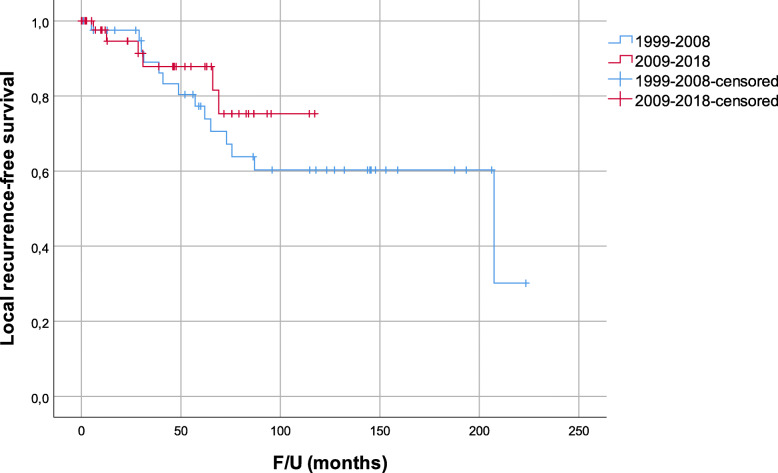
Local recurrence-free survival divided by the period (*p* = 0.876)

By dividing the cohort in two groups, 41 were resected in the first period and 48 in the second. Clinicopathologic characteristics for the entire series and comparison for both periods are presented in Table 2. In the first period, none of the patients died within 90 days after surgery while in the second period 90-day mortality rate was 10.4%. In total, 22 (53.7%) patients from the first and 17 (35.4%) from the second period died. Median follow-up for the first and second period was 95.9 and 46.2 months. The corresponding 5-year OS (Fig. 4) and DSS were 72.8% and 79.6% for patients from the first and 62.9% and 66.4% for patients from the second period. The corresponding 5-year LRFS was 77.3% and 87.8% for the first and second period, respectively (Fig. 5). The differences in OS, DSS, and LRFS between the two periods were not statistically significant.

**Table 2:** Clinicopathologic characteristics of patients for entire series and from first (1999–2008) and second (2009–2018) period

Characteristic	All patients, *n* = 89	First period, *n* = 41	Second period, *n* = 48	*p*
Gender				0.833
Male	47 (52.8%)	21 (51.2%)	26 (54.2%)	
Female	42 (47.2%)	20 (48.8%)	22 (45.8%)	
Age, median (years)	62 (range 24–84)	64 (range 31–82)	62 (range 24–84)	0.922
ASA score				0.108
1	17 (19.1%)	11 (26.8%)	6 (12.5%)	
2	43 (48.3%)	17 (41.5%)	26 (54.2%)	
3	22 (24.7%)	9 (22.0%)	13 (27.1%)	
4	4 (4.5%)	1 (2.4%)	3 (6.3%)	
Unknown	3 (3.4%)	3 (7.3%)	0	
Histologic subtype				0.424
Dedifferentiated liposarcoma	34 (38.2%)	14 (34.1%)	20 (41.7%)	
Well-differentiated liposarcoma	19 (21.3%)	12 (29.3%)	7 (14.6%)	
Leiomyosarcoma	14 (15.7%)	7 (17.1%)	7 (14.6%)	
Solitary fibrous tumor	8 (9.0%)	3 (7.3%)	5 (10.4%)	
Malignant peripheral nerve sheath tumor	2 (2.2%)	0	2 (4.2%)	
Other	12 (13.5%)	5 (12.2%)	7 (14.6%)	
FNCLCC Grade				0.054
I	31 (34.8%)	21 (51.2%)	10 (20.8%)	
II	16 (18.0%)	5 (12.2%)	11 (22.9%)	
III	30 (33.7%)	13 (31.7%)	17 (35.4%)	
Unknown	12 (13.5%)	2 (4.9%)	10 (20.8%)	
Median tumor size (cm)	21 (range 3–80)	24 (range 7–80)	19.5 (range 3–58)	0.403
Radiotherapy				
Neoadjuvant	4 (4.5%)	0	4 (8.3%)	0.059
Adjuvant	7 (7.9%)	5 (12.2%)	2 (4.2%)	0.241
Chemotherapy				
Neoadjuvant	2 (2.2%)	1 (2.4%)	1 (2.1%)	1.0
Adjuvant	2 (2.2%)	2 (4.9%)	0	0.209
Surgical resection margin				0.044
R0	68 (76.4%)	27 (65.9%)	41 (85.4%)	
R1	21 (23.6%)	14 (34.1%)	7 (14.6%)	
R2	0	0	0	
Median time to treatment (days)	27.0 (range 0–181)	16.0 (range 0–65)	35.0 (range 4–181)	< 0.001
Median weight of the specimen (g)	2259 (range 12–32,600)	3450 (range 86–32,600)	2006 (range 12–13,000)	0.087
Stage (AJCC 8th edition)				0.166
1A	1 (1.1%)	0	1 (2.1%)	
1B	42 (47.2%)	23 (56.1%)	19 (39.6%)	
3A	7 (7.9%)	1 (2.4%)	6 (12.5%)	
3B	39 (43.8%)	17 (41.5%)	22 (45.8%)	
Median surgery duration (hours)	7.3 (range 1.3–19.0)	7.5 (range 2–14.5)	7.0 (range 1.3–19)	0.669
Median blood loss (l)	1.0 (range minimal–32)	0.8 (range minimal–32)	1.4 (range minimal–30)	0.853
Resection type				0.266
Tumorectomy	2 (2.2%)	0	2 (2.2%)	
Tumor removed with at least one organ, but not compartmental resection	45 (50.6%)	19 (21.3%)	26 (29.2%)	
Compartmental resection	42 (47.2%)	22 (24.7%)	20 (22.5%)	
Complication rate				0.214
Clavien-Dindo 3a	7 (7.9%)	4 (9.8%)	3 (6.3%)	
Clavien-Dindo 3b	9 (10.1%)	5 (12.2%)	4 (8.3%)	
Clavien-Dindo 4a	3 (3.4%)	1 (2.4%)	2 (4.2%)	
Clavien-Dindo 4b	3 (3.4%)	0	3 (6.3%)	
Clavien-Dindo 5 (90 days)	5 (5.6%)	0	5 (10.4%)	
Median hospital stay after surgery (days)	22.0 (range 2–102)	23.0 (range 10–77)	21.0 (range 2–102)	0.952
Median ICU stay (days)	8.0 (range 0–55)	9.0 (range 4–22)	7.5 (range 0–55)	0.939

*ASA* American Society of Anesthesiologists classification, *FNCLCC* Fédération Nationale des Centres de Lutte Contre Le Cancer, *AJCC* American Joint Committee on Cancer, *ICU* intensive care unit

Nine patients (9.2%, 9/98) diagnosed with primary localized RPS in the study period were not surgically treated because of comorbidities (*n* = 5), old age (*n* = 2), irresectability (*n* = 1), and patient decision (*n* = 1).

## Discussion

Management of sarcoma patients in referral sarcoma centers is organized in different ways. The Sarcoma Policy Checklist was created by European multidisciplinary expert group in 2017 recommending that each country should have at least one designated and accredited center of reference for sarcoma patients and that patients should receive multidisciplinary care delivered by a specialized sarcoma team [6]. Slovenia has a population of 2.1 million. From the referral sarcoma center point of view, this poses a unique situation. Institute of Oncology Ljubljana was established in 1938 and is the only referral sarcoma center in the country. Sarcoma MDT was established in 1975. It currently involves 23 dedicated sarcoma specialists with two specialized sarcoma surgeons, three orthopedic surgeons and a plastic surgeon. At the MDT, we manage all soft tissue and bone sarcoma patients in the country. We are the only institution having facilities for management and treatment. The highest incidence of soft tissue sarcoma patients in Slovenia in the study period was 112, noted in the national registry database in 2015 [7]. According to European Cancer Organisation recommendations, the institution is considered a sarcoma referral center if at least 100 new soft tissue and bone sarcoma patients are treated per year [8]. They also state that sarcoma surgeon should perform at least 2–3 sarcoma operations per month. We operate patients with soft tissue tumors of the extremities, trunk, superficial part of head and neck, retroperitoneum, pelvis, abdominal viscera, and pediatric sarcoma patients at the University Clinical Centre Ljubljana. In total, we perform around 70–80 sarcoma operations per year. Hospital case volume of our institution is about 90 cases per year and surgeon case volume is at least 2 sarcoma operations per month.

Recently, Villano et al. published a multi-institutional analysis of hospital volume-outcome relationship and identified 13 cases of RPS operations per year as a minimum volume threshold for optimal outcome [9]. Institutions meeting this threshold were declared as high volume hospitals (HVH). In our hospital, the average number of resections for primary RPS was 4.5 cases per year in the study period, not meeting the criteria for HVH. However, in the study period, we operated on 28 additional patients with recurrent or residual RPS which are often much more challenging and demanding than the primary ones. Furthermore, factors likely to play a major role in the outcome such as availability of MDT, surgeon case volume and experience, intensive care unit specialists, team for clinical nutrition, interventional radiologists, and others were not accounted for in their analysis. In our hospital, all the expertise mentioned needed for the optimal management of RPS patients is available. Our long-term results are comparable to the largest and most cited series of primary RPS, indicating that lower volume centers may achieve competitive results as long as they have appropriate facilities and expertise. Five-year OS and LRFS for the entire series from our institution were 67.2% and 81.5% and are comparable among other with results of the largest study so far, which included 1007 patients, reported in 2015 from the Transatlantic Retroperitoneal Sarcoma Working Group [10]. Comparison of the outcome data with reports from the literature is shown in Table 3.

**Table 3:** Some reported series of primary retroperitoneal sarcoma

Author	Published	Period	Patients	Median FU (months)	Complete resection %	5-year OS %	5-year LRFS %
Kilkenny et al. [11]	1996	1970–1994	63	*	78	48	*
Lewis et al. [12]	1998	1982–1997	231	28	80	54	59
Stoeckle et al. [13]	2001	1980–1994	145	47	65	49	42
Ferrario et al. [14]	2003	1977–2001	79	41	99	65	43
Hassan et al. [15]	2004	1983–1995	97	36	78	51	56
Van Dalen et al. [16]	2007	1989–1994	143	122	55	39	*
Strauss et al. [17]	2010	1990–2009	200	29	85	68	55
Toulmonde et al. [18]	2014	1988–2008	389	78	100	66	46
Gronchi et al. [10]	2015	2002–2011	1007	58	95	67	74
Our series	-	1999–2018	89	62	100	67	81

*Not applicable

Postoperative complication grade 3a or higher according to Clavien-Dindo classification had 30.3% of patients (Table 2) and 2.2% of patients died within 30 days after the operation. These data could be compared to Transatlantic Retroperitoneal Sarcoma Working Group report published by MacNeill et al. in 2018 where the rate of severe postoperative adverse events was 16.4% and 1.8% of patients died in the early postoperative period [19]. Our results are acceptable, but 90-day mortality rate of 10.4% in the second period was, however, high in our series (Table 1). In the future, we are going to try to adopt the enhanced recovery after surgery protocol to try to improve the results.

Finally, in a series of 89 consecutive patients surgically treated for primary localized RPS at our institution in a period of 20 years, 41 were treated in the first and 48 in the second decade. Comparing clinical and pathologic variables and the management of patients between the two periods, we found no major differences. Only variables that significantly differed were median time to treatment and proportion of R0 resection. Median time to treatment was more than twice as long in the second period. Possible reasons that might explain the difference are higher number of patients transferred directly from a local hospital to the Institute for treatment in the first period (26.8% vs 4.2%), higher number of patients having core needle biopsy in the second period (31.3% vs 2.4%), and a trend towards longer preparation for surgery with parenteral nutrition in the second period (11.5 days vs 8.2 days). The proportion of R0 resections in the second period was 85.4% almost 20% higher than in the first period. Possible reason could be a trend to smaller pathologic specimen. There was a trend towards overrepresentation of higher grade and smaller tumors in the second period, higher proportion of neoadjuvant radiotherapy in the second period, and smaller median weight of the specimen in the second period.

Certainly, there are some limitations to the study. The retrospective nature and a long time span of the study could cause potential selection bias. Low number of patients does not allow a large sample size which could affect the results. Nevertheless, the main strength of the study is to emphasize the crucial role of MDT and sarcoma surgeon in management of sarcoma patients.

In conclusion, our institution fulfills the criteria for a sarcoma referral center representing a low volume center for RPS patients. However, membership in the Transatlantic Australasian Retroperitoneal Sarcoma Working Group gives us the opportunity for education, international collaboration, research, and better understanding of the natural history of RPS in the effort for optimal treatment of RPS patients.

## Conclusion

We are aware that HVH offer best chances for the optimal treatment to RPS patients, but results from our institution show that referral sarcoma centers may achieve very good results in management of these patients, despite not meeting the criteria for HVH, as long as they have MDT, appropriate facilities, and expertise.

## Data Availability

The datasets used and analyzed during the current study are available from the corresponding author on reasonable request.

## Abbreviations

RPS: Retroperitoneal sarcoma
OS: Overall survival
DSS: Disease-specific survival
LRFS: Local recurrence-free survival
DM: Distant metastases
LR: Local recurrence
MDT: Multidisciplinary team
HVH: High volume hospital
